# Complete nucleotide sequence of the *Cryptomeria japonica *D. Don. chloroplast genome and comparative chloroplast genomics: diversified genomic structure of coniferous species

**DOI:** 10.1186/1471-2229-8-70

**Published:** 2008-06-23

**Authors:** Tomonori Hirao, Atsushi Watanabe, Manabu Kurita, Teiji Kondo, Katsuhiko Takata

**Affiliations:** 1 Institute of Wood Technology, Akita Prefectural University, 11-1 Kaieisaka, Noshiro, Akita 016-0876, Japan; 2 Forestry and Forest Products Research Institute, Forest Tree Breeding Center, 3809-1 Ishi, Juo, Hitachi, Ibaraki 319-1301, Japan

## Abstract

**Background:**

The recent determination of complete chloroplast (cp) genomic sequences of various plant species has enabled numerous comparative analyses as well as advances in plant and genome evolutionary studies. In angiosperms, the complete cp genome sequences of about 70 species have been determined, whereas those of only three gymnosperm species, *Cycas taitungensis*, *Pinus thunbergii*, and *Pinus koraiensis *have been established. The lack of information regarding the gene content and genomic structure of gymnosperm cp genomes may severely hamper further progress of plant and cp genome evolutionary studies. To address this need, we report here the complete nucleotide sequence of the cp genome of *Cryptomeria japonica*, the first in the Cupressaceae sensu lato of gymnosperms, and provide a comparative analysis of their gene content and genomic structure that illustrates the unique genomic features of gymnosperms.

**Results:**

The *C. japonica *cp genome is 131,810 bp in length, with 112 single copy genes and two duplicated (*trn*I-CAU, *trn*Q-UUG) genes that give a total of 116 genes. Compared to other land plant cp genomes, the *C. japonica *cp has lost one of the relevant large inverted repeats (IRs) found in angiosperms, fern, liverwort, and gymnosperms, such as *Cycas *and *Gingko*, and additionally has completely lost its *trn*R-CCG, partially lost its *trn*T-GGU, and shows diversification of *acc*D. The genomic structure of the *C. japonica *cp genome also differs significantly from those of other plant species. For example, we estimate that a minimum of 15 inversions would be required to transform the gene organization of the *Pinus thunbergii *cp genome into that of *C. japonica*. In the *C. japonica *cp genome, direct repeat and inverted repeat sequences are observed at the inversion and translocation endpoints, and these sequences may be associated with the genomic rearrangements.

**Conclusion:**

The observed differences in genomic structure between *C. japonica *and other land plants, including pines, strongly support the theory that the large IRs stabilize the cp genome. Furthermore, the deleted large IR and the numerous genomic rearrangements that have occurred in the *C. japonica *cp genome provide new insights into both the evolutionary lineage of coniferous species in gymnosperm and the evolution of the cp genome.

## Background

Since the first reports of the complete nucleotide sequences of the tobacco [1] and liverwort [2] chloroplast (cp) genomes, a number of other land plant cp genomic sequences have been determined. These complete cp genomic sequences have enabled various comparative analyses, including phylogenetic studies, that are based on these data [3-7]. In contrast, however, the complete cp genome nucleotide sequences of only three gymnosperm species, *Cycas taitungensis *[8], *Pinus thunbergii *[9], and *Pinus koraiensis *[10] have been determined.

The cp genomes of gymnosperms, especially in coniferous species, have distinctive features compared with those of angiosperms, including paternal inheritance [11-17], relatively high levels of intra-specific variation [18-21], and a different pattern of RNA editing [22]. Generally, the cp genomes of angiosperms range in size from 130 to 160 kb, and contain two identical inverted repeats (IRs) that divide the genomes into large (LSC) and small single copy (SSC) regions. The relative sizes of these LSC, SSC and IRs remain constant, with both gene content and gene order being highly conserved [23,24]. On the other hand, the relative sizes of the gymnosperm IRs vary significantly among taxa [25-27]; for example, the IRs of *Ginkgo biloba *are 17 kbp [28], those of *Cycas taitungensis *are 23 kbp [8], whereas those of *Pinus thunbergii *are very short, at just 495 bp [9,29]. It has been suggested that, like *P*. *thunbergii*, some coniferous species also lack the large IRs that exist in other gymnosperms [25,26,30,31]. This lack of IRs is considered to have preceded the extensive genomic rearrangements of the conifer cp genome [26]. Steane [32] compared the complete cp genome of *Eucalyptus globulus *with that of other angiosperm taxa and *P. thunbergii*, and found that the cp genome of *P. thunbergii *was arranged very differently to that of angiosperms. However, there is only limited information available about the cp genomic sequences of coniferous species, with the complete cp genome nucleotide sequences of only two species of pine, *Pinus thunbergii *[9] and *Pinus koraiensis *[10] in the family Pinaceae, having been determined. The cp genomes of these two pine species were very similar in terms of both gene content and gene order and so provided little information about the complexity of the conifer cp genome.

In previous phylogenetic studies, of the four extant gymnosperm groups (Cycads, Conifers, Ginkgoales, and Gnetales), the conifers were considered to be divisible into two distinct groups; a Pinaceae group and a group consisting of five other families (Cupressaceae sensu lato, Taxaceae, Podocarpaceae, Araucariaceae, and Sciadopityaceae) [33,34]. The cp nucleotide sequences from this five member group, excluding the Pinaceae group, can provide interesting information about the conifer cp genome, not only in terms of genome structure but also concerning their evolutionary history. Despite the lack of complete cp genome sequences from any family member of the Cupressaceae sensu lato, Tsumura *et al*. [27] suggested, on the basis of physical maps and Southern hybridization analyses, that the cp genome of *Cryptomeria japonica *differs from that of other land plants, including pine species, in terms of genome size and gene order as well as in the absence of the large IRs. Thus, the complete cp genome sequence of *C. japonica *would drastically increase our understanding of the divergence of coniferous cp genome structures and gene content, and additionally clearly identify the differences with the Pinaceae group.

There are two particular questions that need to be addressed using the complete cp genome sequence of *C. japonica*: (1) how different is the *C. japonica *cp genome from those of other plants, including gymnosperms, and (2) is the loss of the large IRs involved with the instability and diversification of the cp genome, especially between coniferous groups? To respond to these questions, we present in this paper the complete nucleotide sequence of the cp genome of *C. japonica *[DDBJ: AP009377], and compare its overall gene content and genomic structure with those of two other angiosperms (*Eucalyptus globulus *and *Oryza sativa*), a liverwort (*Marchantia polymorpha*), a fern (*Adiantum capillus*), and two gymnosperms (*Cycas taitungensis *and *Pinus thunbergii*).

## Results and Discussion

### General characteristics of the *C. japonica *cp genome

**Figure 1:**
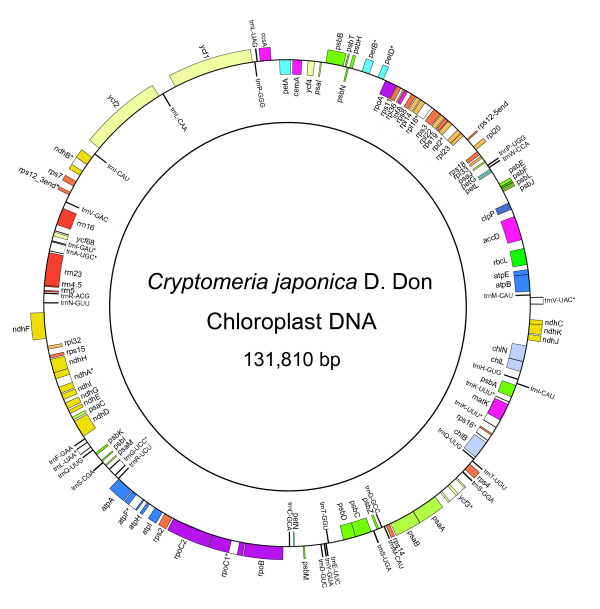
**Gene organization of the *C. japonica *chloroplast genome (see Table 1)**. Genes shown outside the circle are transcribed clockwise, while those located inside are transcribed counter-clockwise. Intron-containing genes are indicated by asterisks. Red boxes, ribosomal RNA genes; black boxes, transfer RNA genes; light orange boxes, large subunit of ribosomal protein genes; dark orange boxes, small subunit of ribosomal protein genes; dark purple boxes, DNA dependent RNA polymerase genes; dark green boxes, *rbc*L gene; yellowish-green boxes, subunits of photosystem I genes; green boxes, subunits of photosystem II genes; light blue boxes, subunits of cytochrome genes; dark blue boxes, subunits of ATP synthase genes; light yellow boxes, ORF genes; dark yellow boxes, subunits of NADH dehydrogenase genes; light purple boxes, chlorophyll biosynthesis genes. The pseudogene is indicated by ψ (pseudo-).

The total size of the *C. japonica *cp genome was determined to be 131,810 bp, which is larger than the cp genomes of both *P. thunbergii *(119,707 bp) and *M. polymorpha *(121,024 bp), but smaller than those of *A. capillus *(150,568 bp), *E. globulus *(160,286 bp), and *C. taitungensis *(163,403 bp), and approximately the same size as that of *O. sativa *(134,558 bp). This size is only slightly smaller than that previously estimated by RFLP southern hybridization analysis [27]. The large IR region, which is found in other land plants except *Pinus*, could also not be observed in the *C. japonica *cp genome, and so we were unable to define the large (LSC) and small (SSC) single copy regions in this genome. A total of 116 genes were identified in the *C. japonica *cp genome, of which 112 genes were single copy and two genes, *trn*I-CAU and *trn*Q-UUG, were duplicated and occurred as inverted repeat sequences. There were four ribosomal RNA genes (3.5%), 30 individual transfer RNA genes (25.9%), 21 genes encoding large and small ribosomal subunits (18.1%), four genes encoding DNA-dependent RNA polymerases (3.5%), 48 genes encoding photosynthesis-related proteins (41.4%), and 9 genes encoding other proteins, including those with unknown functions (7.8%). Among the 112 single copy genes, 17 genes contained introns, and three genes, *clp*P, *trn*T-GGU, and *ycf*68, were identified as pseudogenes. The locations of the genes and pseudogenes are shown in Figure 1 (gene map) and Table 1 (gene content). The *C. japonica *cp genome has an AT content of 64.6%, which is higher than those of *A. capillus *(58.0%), *C. taitungensis *(60.5%), *O. sativa *(61.0%), and *P. thunbergii *(61.2%), similar to that of *E. globulus *(63.4%), but lower than that of *M. polymorpha *(71.2%).

**Table 1:** List of genes found in *C. japonica *chloroplast genome (see Figure 1)

Category for genes	Group of gene	Name of gene					
Self replication	Ribosomal RNA genes	*rrn *16	*rrn *23	*rrn *5	*rrn *4.5		
	Transfer RNA genes	*trn *A-UGC *	*trn *C-GCA	*trn *D-GUC	*trn *E-UUC	*trn *F-GAA	*trnf *M-CAU
		*trn *G-GCC	*trn *G-UCC *	*trn *H-GUG	*trn *I-CAU × 2	*trn *I-GAU *	*trn *K-UUU *
		*trn *L-CAA	*trn *L-UAA *	*trn *L-UAG	*trn *M-CAU	*trn *N-GUU	*trn *P-GGG
		*trn *P-UGG	*trn *Q-UUG × 2	*trn *R-ACG	*trn *R-UCU	*trn *S-GCU	*trn *S-UGA
		*trn *S-GGA	*trn *T-UGU	*trn *V-GAC	*trn *V-UAC *	*trn *W-CCA	*trn *Y-GUA
	Small subunit of ribosome	*rps *2	*rps *3	*rps *4	*rps *7	*rps *8	*rps *11
		*rps *12*	*rps *14	*rps *15	*rps *16 *	*rps *18	*rps *19
	Large subunit of ribosome	*rpl *2 *	*rpl *14	*rpl *16 *	*rpl *20	*rpl *22	*rpl *23
		*rpl *32	*rpl *33	*rpl *36			
	DNA dependent RNA polymerase	*rpo *A	*rpo *B	*rpo *C1 *	*rpo *C2		
	Translational initiation factor	*inf *A					
Genes for photosynthesis	Subunits of photosystem I	*psa *A	*psa *B	*psa *C	*psa *I	*psa *J	*psa *M
	Subunits of photosystem II	*psb *A	*psb *B	*psb *C	*psb *D	*psb *E	*psb *F
		*psb *H	*psb *I	*psb *J	*psb *K	*psb *L	*psb *M
		*psb *N	*psb *T	*psbZ*			
	Subunits of Cytochrome	*pet *A	*pet *B *	*pet *D *	*pet *G	*pet *L	*petN*
	Subunits of ATP synthase	*atp *A	*atp *B	*atp *E	*atp *F *	*atp *H	*atp *I
	Large subunit of Rubisco	*rbc *L					
	Chlorophyll biosynthesis	*chl *B	*chl *N	*chl *L			
	Subunits of NADH dehydrogenase	*ndh *A*	*ndh *B *	*ndh *C	*ndh *D	*ndh *E	*ndh *F
		*ndh *G	*ndh *H	*ndh *I	*ndh *J	*ndh *K	
Other genes	Maturase	*mat *K					
	Envelop membrane protein	*cemA*					
	Subunit of Acetyl-CoA-carboxylase	*acc*D					
	c-type cytochrome synthesis gene	*ccsA*					
Genes of Unknown function	Conserved Open Reading Frames	*ycf *1	*ycf *2	*ycf *3 *	*ycf *4		
Pseudogenes	Pseudogene	Pseudo-*clp*P	Pseudo-*trn *T-GGU		Pseudo-*ycf *68		

* Genes containing introns.

### A marked difference in gene content between gymnosperms including *C. japonica*

There are marked differences in several genes between gymnosperms, even though the *C. japonica *cp genome shares several common features with other plants, and some of these are described below. For example, there is considerable difference in gene content between *C. japonica *and *P. thunbergii*; the 11 intact *ndh *(NADH dehydrogenase) genes found in *C. japonica*, as well as in five other plants, are absent from *P. thunbergii *[9]. The loss of these *ndh *genes is thought to be due to specific mutations in the *Pinus *cp genome.

**Figure 2:**
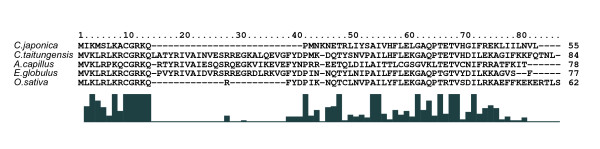
**Amino acid sequences of the *rps*16 genes from five plant cp genomes, including *C. japonica***. The histogram below the sequences represents the degree of similarity. Peaks indicate positions of high similarity, and valleys positions of low similarity. Numbers at the C-terminal ends indicate the length of the amino acid sequences in each species.

Another functional gene, *rps*16, which encodes a small ribosomal subunit, is found in the angiosperms, *E. globulus *and *O. sativa*, in the fern, *A. capillus*, and in gymnosperms, *C. taitungensis *and *C. japonica *(Figure 2). However, the location of *rps*16 is halfway between the *trnK*-UUU and *chl*B genes in the cp genome of gymnosperms, and halfway between *mat*K and *chl*B, and between the *trn*K-UUU and *trn*Q-UUG genes in fern and angiosperms, respectively. In contrast, *rps*16 is completely absent from the *M. polymorpha *and *P. thunbergii *[29,35] cp genomes, in addition to a large number of unrelated taxa of land plants, including *Connarus*, *Epifagus*, *Eucommia*, *Fugus*, *Krameria*, *Linum*, *Malpighia*, *Passiflora*, *Securidaca*, *Turnera*, *Viola*, *Adonis*, *Medicago*, *Selaginella *[36-41]. Doyle *et al*. [38] postulated the functional transfer of *rps*16 from the chloroplast to the nucleus in order to explain the absence of this gene in such a large number of unrelated taxa of land plants. Similarly, the loss of *rps*16 and its functional transfer to the nucleus might have occurred independently in gymnosperms, especially in coniferous species.

The *trn*P-GGG and *trn*R-CCG genes are considered to be pseudogenes, possibly relics of plastid genome evolution in gymnosperms and moss [22,42,43]. The *trn*P-GGG gene is found in *C. japonica*, as well as in the two gymnosperms, *P. thunbergii *and *C. taitungensis*, in the liverwort, *M. polymorpha*, and in the fern, *A. capillus*, but not in angiosperm cp genomes. The gene is also found in *Gnetum *and *Ginkgo *of gymnosperms [8], suggesting that this is a relic gene in a large number of gymnosperms. In contrast, the *trn*R-CCG gene, which is found in *P. thunbergii*, *C. taitungensis*, *M. polymorpha*, and *A. capillus*, is absent from the *C. japonica *and angiosperm cp genomes, suggesting that *trn*R-CCG is not conserved in all gymnosperm cp genomes and might have been completely lost in taxa, such as Cupressaceae sensu lato, that have relatively recently diverged during the long evolutionary history of plants.

**Figure 3:**
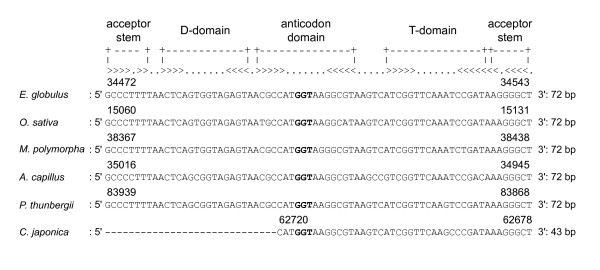
**Nucleotide sequences of the *trn*T-GGU genes of six land plant cp genomes, including *C. japonica***. The *trn*T-GGU gene is missing from the *C. taitungensis *genome and is too short to form a secondary structure in *C. japonica*. The bold characters show the anti-codon GGU. The position of the *trn*T-GGU gene in each cp genome is shown above each sequence. The secondary structure of the *trn*T-GGU gene is described at the top.

The tRNA gene, *trn*T-GGU, in the *C. japonica *cp genome contains only 43 bp of its 3' end and was therefore too short to form its complete secondary structure (Figure 3). Furthermore, this *trn*T-GGU gene occurs as a single copy gene in the cp genomes of *A. capillus*, *M. polymorpha*, *E. globulus*, and *O. sativa*, is present as two copies in *P. thunbergii*, but is completely missing from the *C. taitungensis *cp genome. In *Pelagonium*, the loss of *trn*T-GGU from its cp genome has been considered to be associated with genomic rearrangements [44]. Although this relationship is considered further below, the duplication or incomplete lost of tRNA genes in *P. thunbergii *and *C. japonica *is also thought to be associated with genome rearrangements. However, the question remains as to why the *trn*T-GGU of *C. taitungensis *is completely lost despite the fact that no genomic rearrangements were found in comparison with standard cp genomes, such as of *E. globulus*.

### Diversification of genes in the *C. japonica *cp genome

**Figure 4:**
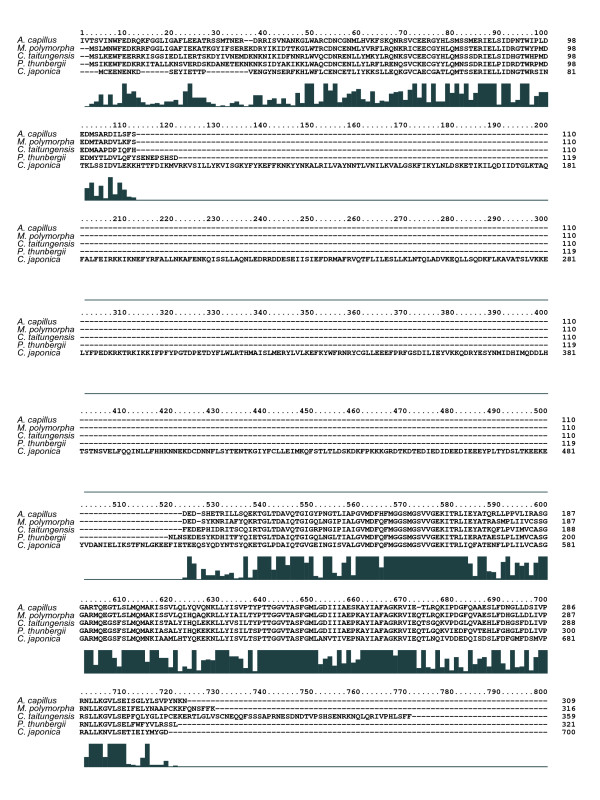
**Alignment of amino acid sequences of the *acc*D gene in five land plant cp genomes**. The histogram indicates the degree of similarity (see Figure 2). The number on the right indicates the length of the *acc*D reading frame in each cp genome. The amino acid length of the *acc*D gene product in *C. japonica *is approximately twice that of the other five plant cp genomes.

The *acc*D gene, which encodes acetyl-CoA-carboxylase (ACCase), is found in the cp genomes of all seven plants analyzed in this study, however, their reading frame lengths vary considerably. The reading frame length of the *C. japonica *cp genome is 700 codons, which is larger than that of *A. capillus *(309 codons), *M. polymorpha *(316 codons), *P. thunbergii *(321 codons), and *C. taitungensis *(359 codons) (Figure 4). The alignments do not include those of the angiosperms, *E. globulus *(490 codons), and *O. sativa *(106 codons), because of the complicated nature of the alignments. In monocot angiosperms, the *accD *reading frame length is reduced from 106 codons in *O. sativa *to zero in *Z. mays*, and this reduction is considered to be the cause of *acc*D loss in monocot species [45]. In contrast to this reduction, the *acc*D reading frame in coniferous species, especially in Cupressaceae sensu lato including *C. japonica*, may have diversified in an increasing direction.

**Figure 5:**
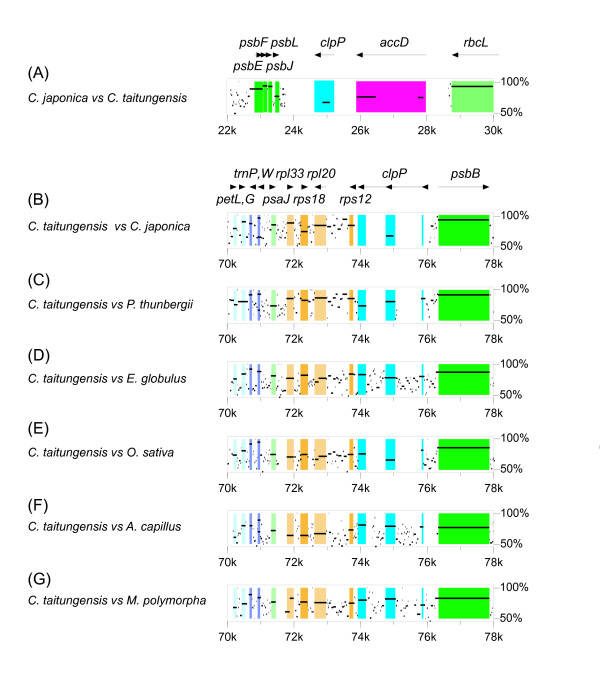
**Percentage identity plots and gene order surrounding the *clp*P gene**. Gene identities between *C. japonica *and *C. taitungensis *(A), and between *C. taitungensis *and six other plants including *C. japonica *(B-G) are shown by MultiPipMaker. The directions of arrows indicate the transcribed sense/antisense strands. The colored boxes show the group of genes and the relevant coding region of each gene. Mutual comparisons of the *clp*P gene between *C. japonica *and *C. taitungensis *(A, B), show the first and third exon are completely absence in the *C. japonica *cp genome. For the gene order surrounding the *clp*P gene, the *clp*P to *rpl*20 gene order is extremely conserved to be co-transcribed in the cp genomes of all the land plants of this study (see Figure 7 for the detailed gene order). On the other hand, the *clp*P gene in the *C. japonica *cp genome is found halfway between the *psb*J and *acc*D gene, and is clearly not co-transcribed with the *rps*12 and *rpl*20 genes.

The *clp*P gene, which encodes a proteolytic subunit of the ATP-dependent Clp protease, is found intact in the cp genomes of the six land plants, *C. taitungeinsis, E. globulus, A. capillus*, and *M. polymorpha*, with three exons and two introns, and in the *P. thunbergii *and *O. sativa *cp genomes with no introns [22]. However, in the *C. japonica *cp genome, only the second exon of the gene remains and so it occurs as a pseudogene. Furthermore, the *clp*P gene is co-transcribed with the 5'-end of the *rps*12 gene and the *rpl*20 gene (*M. polymorpha*; [46], *P. contorta*; [47], *O. sativa*; [48]), so that the *clp*P to *rpl*20 gene order is extremely conserved in the cp genomes of all the land plants of this study. However, the *clp*P gene in the *C. japonica *cp genome is found halfway between the *psb*J and *acc*D genes, and is clearly not co-transcribed with the *rps*12-5'end and *rpl*20 genes (Figure 5). As the loss of function of the *clp*P gene in the *Adonis annua *cp genome is thought to be due to genome rearrangements (inverted mutations) [39], it is possible that genome rearrangements are also the reason why *clp*P is a non-functional pseudogene in the *C. japonica *cp genome, as discussed further below.

**Figure 6:**
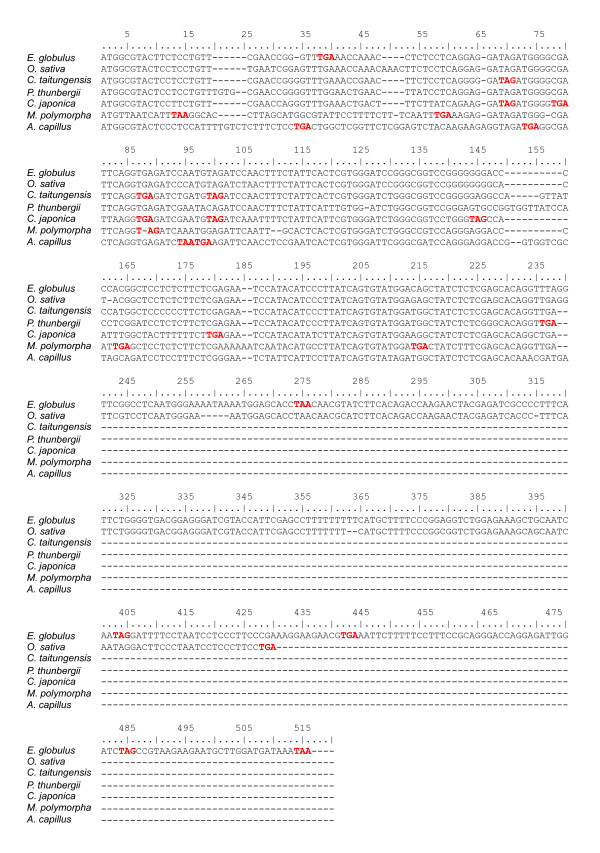
**Alignment of the *ycf*68 regions of seven land plant cp genomes**. Sequences of the *ycf*68 region of *P. thunbergii *and *O. sativa *were obtained from databases of each complete cp genome sequence, and relevant regions of the other plants were obtained by alignment with that of *P. thunbergii *or *O. sativa*. Codons highlighted in red represent stop codons.

Although four major *ycf *genes have been partially characterized in the cp genomes of other land plants, their precise functions remain unclear to date. Four *ycf *genes, *ycf*1, *ycf*2, *ycf*3, and *ycf*4, were also identified in the *C. japonica *cp genome. The highly conserved *ycf*3 and *ycf*4 are believed to be involved in the formation of photosystem I in *Chlamydomonas reinhardtii *[49]. The deduced amino acid sequences of the *ycf*3 and *ycf*4 products show 81–96% and 71–76% sequence identity, respectively, with their homologues in other land plants. In contrast, *ycf*1 and *ycf*2 show considerable divergence relative to other land plants, with their deduced proteins having only 24–54% (partially 54% identity with that of *P. thunbergii*) and 25–37% sequence identity, respectively, with their homologues in other land plants. The two divergent *ycf*1 and *ycf*2 genes are thought to be involved in cellular metabolism or to play a structural role in plastids [50]. Both the maize and rice cp genomes lack these two reading frames [45,51], and the results from the present comparative analysis show that there are no regions homologous to *ycf1 *and *ycf2 *in *C. japonica*. Furthermore, although the y*cf*68 gene of *C. japonica *shows 63% identity to that of *P. thunbergii*, the *C. japonica ycf*68 may not encode a protein. The *ycf*68 sequence, which occurs in the *trn*I-GAU intron, could represent a functional protein encoding gene in rice, corn, and *Pinus*, although alignments of the *ycf*68 region in 14 angiosperms revealed that, in the majority of cases, it contained numerous frameshifts and stop codons [52]. Similarly, we found numerous frameshifts and stop codons in the *ycf*68 region, although the *C. japonica *and *C. taitungensis ycf*68 regions have a comparatively high level of homology with that of *P. thunbergii *(Figure 6).

### Loss of large IR region within coniferous cp genomes

**Figure 7:**
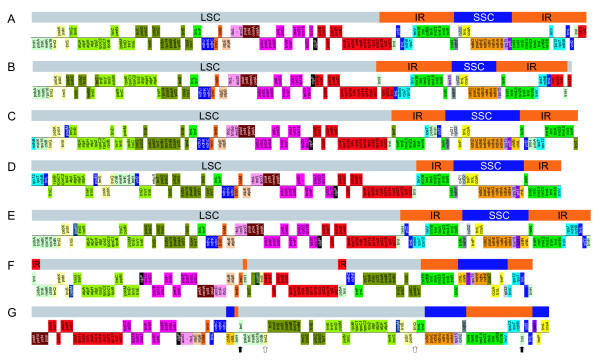
**Gene order and cp genomic architecture of the seven land plant species, including *C. japonica***. Each colored gene segment shows the same gene order region among the seven land plants cp genomes. Gray, blue and orange boxes for each gene order show the relevant regions of LSC (Large Single Copy), SSC (Small Single Copy) and IR (Inverted Repeat) regions in the *E. globulus *(A), *O. sativa *(B), *A. capillus *(C), *M. polymorpha *(D), and *C. taitungensis *(E) cp genomes, respectively. In the *P. thunbergii *(F) and *C. japonica *(G) cp genomes, gray, blue and orange boxes show the relevant regions of the SSC and IR regions of the *C. taitungensis *cp genome. Red boxes in the *P. thunbergii *cp genome show the defined IR in the *P. thunbergii *cp genome. In the *C. japonica *cp genome, the black and white arrows show duplicated genes; *trn*I-CAU (black arrows), *trn*Q-UUG (white arrows).

Figure 7 details the gene order and locations of the LSC, SSC, and IRs of the cp genomes of the seven land plants, *E. globulus *(A), *O. sativa *(B), *A. capillus *(C), *M. polymorpha *(D), *C. taitungensis *(E), *P. thunbergii *(F), and *C. japonica *(G). The *C. japonica *and *P. thunbergii *cp genomes have lost one of the large inverted repeats (IRs) that are found in the cp genomes of other plants. When compared to the *C. taitungensis *cp genome (Figure 7E), which has a large IR region, the corresponding IR of the *C. japonica *cp genome was divided into two segments, and the relevant SSC region was divided into three segments (Figure 7G). Similarly, in the *P. thunbergii *cp genome, the relevant IR region was divided into three segments (Figure 7F). Although the IR of *P. thunbergii*, which is 495-bp in length, contains a duplicated *trn*I-CAU gene and a partial *psb*A gene (red boxes in Figure 7F), presumably due to incomplete loss of the large IR [29], the IRs of *Pinus *cp genomes are thought to be structurally different from those of other plants, being composed of two or more genes including the *trn*I-CAU gene. There are two pairs of short inverted repeats in the *C. japonica *cp genome, consisting of 284-bp and 114-bp inverted repeats containing duplicated *trn*Q-UUG (white arrows in Figure 7G) and *trn*I-CAU (black arrows in Figure 7G) genes, respectively. Based on the defined IRs of the *Pinus *cp genome, the residual IR of *C. japonica *may be the 114-bp inverted repeat containing the duplicated *trn*I-CAU gene. However, it is structurally different from the IRs of other plants that contain several duplicated genes in their cp genomes.

### Structural differences between cp genomes of *C. japonica *and other land plants

**Figure 8:**
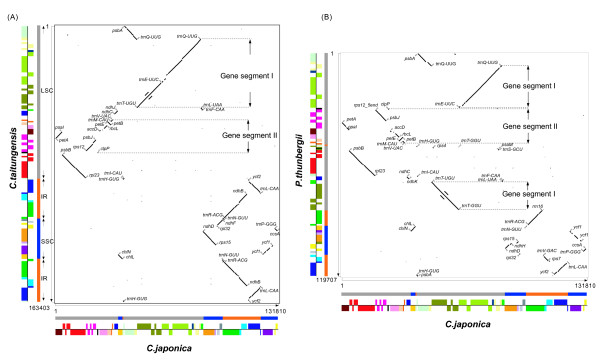
**Harr plot analyses comparing the cp genome of *C. japonica *with those of *C. taitungensis *and *P. thunbergii***. Each dotplot shows the positions where 45 out of 50 nucleotides match in the two sequences. The plot analysis was carried out using Pipmaker software. Sequences along the Y-axis are set from the top to the bottom, and along the X-axis are from left to right. Relative lengths of sequences are shown to the side and below the boxes. The colored gene segments along the X- and Y-axes correspond with common gene units of the seven cp genomes (shown in Figure 7). At the expected endpoint of inversion or translocation mutation, the gene name is attached based on the X-axis (*C. japonica *cp genome). The pseudogene is indicated by ψ (pseudo-). The representative inversion and translocation are represented by gene segment I (the *trn*T-UGU to *trn*Q-UUG of *C. japonica *cp genome) and gene segment II (thr *trn*V-GAC to pseudo-*clp*P of *C. japonica *cp genome). The detailed comparisons of the gene segment I and II are shown in the following Figure 9 and Figure 10.

In addition to the loss of the large IR, genome rearrangements appear to have played an important role in the evolution of the coniferous cp genome. Harr-plot analyses also indicate that the cp genome of *C. japonica *has lost its large IR and that its structure differs significantly from that of the cp genomes of the other six plants in terms of gene order. We estimated the minimum rearrangements via inversions in pairwise comparisons of cp genomes in order to determine the structural differences between cp genomes (Table 2), even though inversions may not be the only mutational events causing gene order changes in the cp genome. A minimum of five inversions would be required to transform the gene structure of the gymnosperm *C. taitungensis *cp genome into that of the angiosperm *E. globulus *cp genome (Table 2, additional file 1A). In contrast, many genome rearrangements have occurred in the cp genomes of coniferous species within gymnosperms; we found that deletion of the large IR and a minimum of 12 inversions would be required to transform the gene structure of the *C. taitungensis *cp genome into that of *C. japonica *(Table 2, Figure 8A), and that deletion of the large IR and a minimum of seven inversions would be required to transform the gene structure of the *C. taitungensis *cp genome into that of *P. thunbergii *(Table 2, additional file 1B). Furthermore, it is interesting to note that 15 inversions would be required to transform the gene structure of *C. japonica *into that of *P. thunbergii *(Table 2, Figure 8B).

**Table 2:** Minimum rearrangements via inversions in pairwise comparisons of seven chloroplast genomes

comparison species	large inversion*	one gene inversion**	loss of a large IR	total inversion
*C. japonica *vs *P. thunbergii*	11	4	-	15
*C. japonica *vs *C. taitungensis*	8	4	1	13
*C. japonica *vs *A. capillus*	16	4	1	21
*C. japonica *vs *M. polymorpha*	12	3	1	16
*C. japonica *vs *E. globulus*	12	4	1	17
*C. japonica *vs *O. sativa*	16	3	1	20
				
*P. thunbergii *vs *C. taitungensis*	6	1	1	8
*P. thunbergii *vs *A. capillus*	13	2	1	16
*P. thunbergii *vs *M. polymorpha*	7	1	1	9
*P. thunbergii *vs *E. globulus*	8	2	1	11
*P. thunbergii *vs *O. sativa*	12	1	1	14
				
*C. taitungensis *vs *A. capillus*	9	1	-	10
*C. taitungensis *vs *M. polymorpha*	3	0	-	3
*C. taitungensis *vs *E. globulus*	4	1	-	5
*C. taitungensis *vs *O. sativa*	8	0	-	8
				
*A. capillus *vs *M. polymorpha*	8	1	-	9
*A. capillus *vs *E. globulus*	9	2	-	11
*A. capillus *vs *O. sativa*	13	1	-	14
				
*M. polymorpha *vs *E. globulus*	3	1	-	4
*M. polymorpha *vs *O. sativa*	7	0	-	7
				
*E. globulus *vs *O. sativa*	4	1	-	5

* large inversions indicate an inversion event of the segment composed of two or more genes.

** one gene inversion indicates the inversion event of one gene.

The large IR is thought to stabilize the cp genome against major structural rearrangements [53-55]. Among angiosperm species, structural changes in the cp genome have occurred within tribes of the legume family (Fabaceae), which have also lost their IR, and so it appears that most genomes that have lost their IRs have undergone more rearrangements than those that have not [53,56]. With respect to other conifers, it has been shown that Douglas fir (*Pseudotsuga menziesii*) and radiata pine (*Pinus radiata*) lack the large IR, and that both of these conifer genomes have undergone a greater number of rearrangements relative to ferns, angiosperms, and even *Ginkgo*, a gymnosperm [26]. The differences in genome structure between *C. japonica *and other land plants, including pines, strongly confirms that the presence of large IRs plays a role in the structural stability of the cp genome.

Tsumura *et al*. [27] suggested that the cp genome structure of *C. japonica *differs significantly from that of pine species, implying that independent changes have occurred and that no simple evolutionary path can be determined. In fact, phylogenetic studies have revealed the significant divergence of Coniferales [33,34], with a phylogenetic tree using the *rbc*L gene in one of these studies indicating that *C. japonica *(Cupressaceae sensu lato) and pine species (Pinaceae) are not very closely related and are in fact located in different clade (additional file 2 in this study). In a study of 18 Campanulaceae species, Cosner *et al*. [57] suggested that data regarding cp genome rearrangements were useful for inferring phylogenetic relationships, and actually found that the results of analysis using gene order closely paralleled the results of phylogenetic analysis using Internal Transcribed Spacer (ITS) and *rbc*L sequence data. Hence, data on rearrangements in the conifer cp genome might reflect phylogenetic relationships and serve as a new evolutionary-related parameter. Furthermore, insights obtained from these studies will provide a clearer detail of the process of cp genome evolution. However, in order to better understand the complex changes in the cp genome structure that have occurred during the long process of evolution, data on the cp genomes of other coniferous taxa, such as Taxaceae, Sciadopityaceae, Podocarpaceae, and Araucariaceae will be required.

### The vestiges of genome rearrangement within the *C. japonica *cp genome

**Figure 9:**
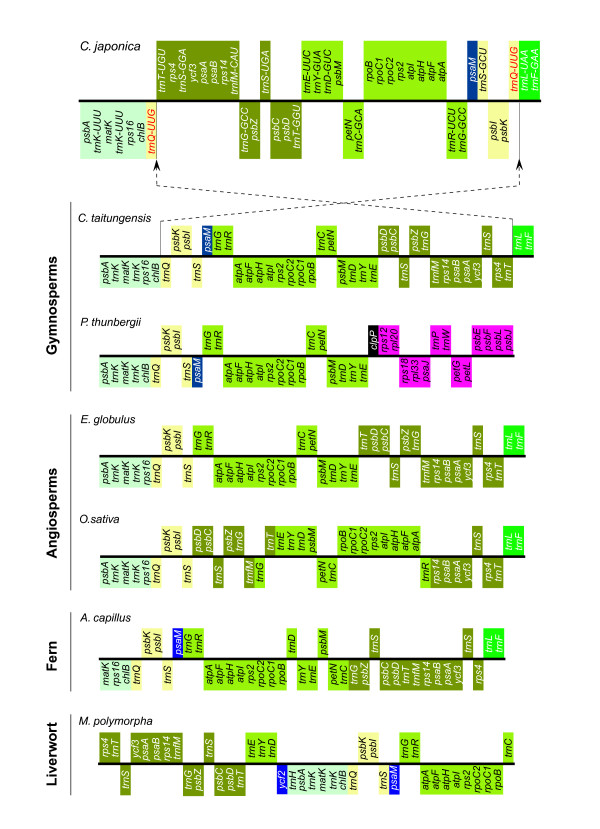
**Expected inversion event in the *C. japonica *cp genome**. The expected inversion corresponds with the gene segment I in Figure 8. Genes are represented by boxes extending above or below the base-line depending on the direction of transcription. The colored boxes indicate the same gene units among the seven cp genomes, including *C. japonica*. The tRNA anti-codon is abbreviated in the six plant cp genomes excluding *C. japonica*. The character highlighted in red represents the duplicated *trn*Q-UUG in the *C. japonica *cp genome. The pseudogene is indicated by ψ (pseudo-).

Dispersed repetitive sequences with duplicated tRNA genes have been reported in the cp genomes of other *Pinus *species [58,59], and are associated with numerous DNA rearrangements, including the loss of IRs [59]. In addition, intact tRNA genes and dispersed repeats that are segments of tRNA sequences have a relationship with the inversion endpoints [23,60-62], although not all inversion borders are near tRNA genes [61]. In this study, the gene order between *psb*A or *mat*K and *trn*S-GCU in the cp genome of six other plants examined was highly conserved, whereas that of the *C. japonica *cp genome differed significantly from these six plants (Figure 9). Assuming a *C. taitungensis*-like ancestral cp genome, we postulate an inversion event, which occurred at the segment from *trn*Q-UUG to *trn*T-UGU, to explain the cause of the duplicated *trn*Q-UUG gene (gene segment I in Figure 8A, and Figure 9).

Within the large inversion from *trn*T-UGU to *trn*Q-UUG, we found another vestige of the genome rearrangement. As mentioned above, the incomplete loss of *trn*T-GGU (halfway between *trn*E-UUC and *psb*D in the *C. japonica *cp genome, Figure 9) from the *C. japonica *cp genome may have been the result of genome rearrangement. In grasses, such as *O. sativa*, it has been suggested that rearrangements in the region surrounding *trn*T-GGU were derived from two independent inversions [49,61,62]. In the *A. capillus *cp genome, the segment from *trn*T-GGU to *trn*G-GCC is inverted when compared to that of *E. globulus*. In the *P. thunbergii *cp genome, a translocation and inversion event occurred at the segment from *trn*T-GGU to the pseudogene *ndh*C (as indicated within gene segment I in additional file 1B). It is worth noting that *trn*T-GGU is located at the borders of the sites of the genome rearrangements. Although the rearrangement associated with *trn*T-GGU was not found in the *C. japonica *cp genome when compared to that of *E. globulus*, the incomplete loss of *trn*T-GGU in the *C. japonica *cp genome suggests the possibility of a re-inversion event.

**Figure 10:**
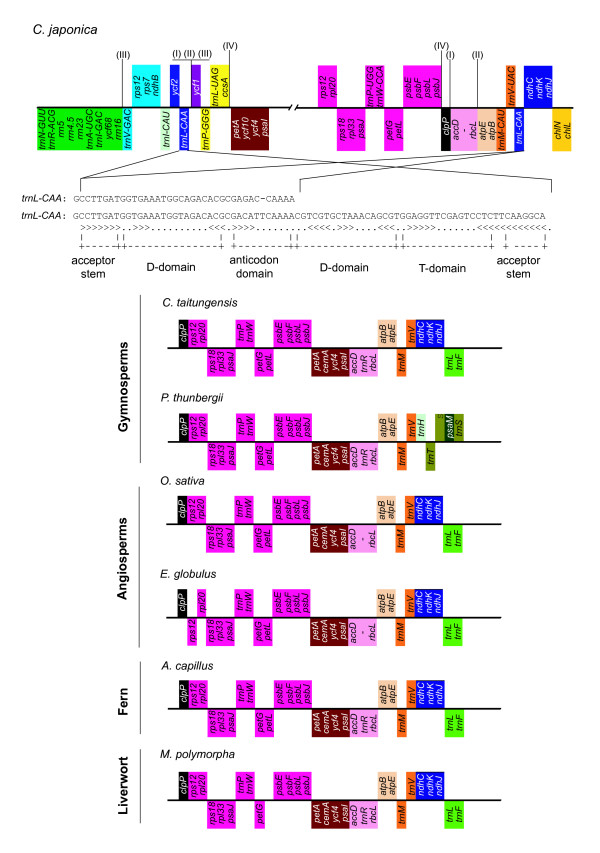
**Expected inversion or translocation endpoints and dispersed repetitive sequences of the *C. japonica *cp genome**. The expected inversion corresponds with gene segment II in Figure 8. Genes are represented by boxes extending above or below the base-line depending on the direction of transcription. The colored boxes indicate the same gene units among the seven cp genomes including *C. japonica*. The number above the gene segments of the *C. japonica *cp genome correspond with positions of each repetitive sequence, and the character (similarity, length, repeat type, location, and sequence) of each repetitive sequence is shown in additional file 3. The *trn*L-CAA gene sequence of the *C. japonica *cp genome is shown with its secondary structure. The *trn*L-CAA with ψ (pseudo-) is incomplete in length to form its secondary structure.

Furthermore, the gene order between the *clp*P and *trn*V-UAC genes is extremely conserved among the six other land plants studied, whereas that of the *C. japonica *cp genome is significantly different (Figure 10). Within the *trn*N-GUU to *chl*L gene segment of the *C. japonica *cp genome, we identified three inverted repeats and one direct repeat which were 50 bp or longer and showed a sequence identity of at least 90%, together with a duplicated partial *trn*L-CAA gene (repetitive sequences of I-IV in Figure 10 and additional file 3). We infer that these repetitive sequences are associated with the inversion and translocation events, because the repetitive sequences were not observed in the other six plant cp genomes and they coincided with rearrangement endpoints that were significantly different from the six other plant cp genomes. However, it is difficult to unequivocally establish the process of genome rearrangement in the *C. japonica *cp genome based solely on the positional information of these repetitive sequences. In particular, we cannot infer why several repetitive sequences are concentrated within the region between *trn*L-CAA and *ycf*1 (repetitive sequences of I-III in Figure 10 and additional file 3).

**Figure 11:**
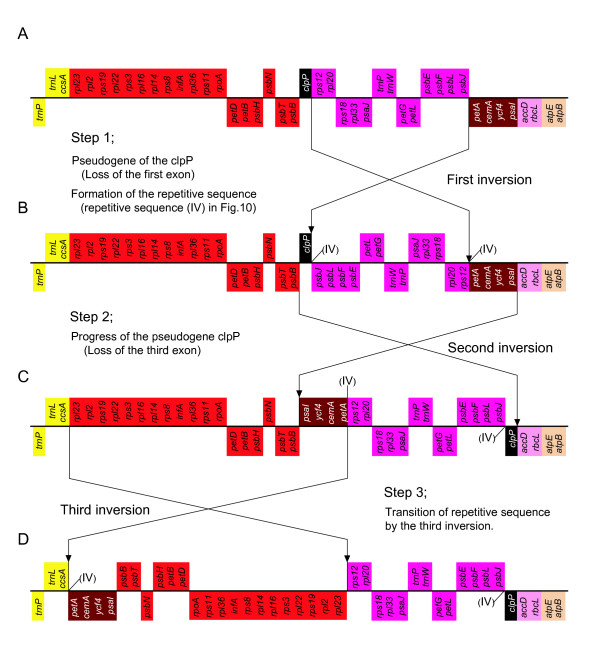
**A three-step model for genome rearrangement with the *clp*P pseudogene in *C. japonica *cp genome**. (A) the hypothesized ancestral cp genome of *C. japonica*; (B), (C) the hypothesized genome rearrangement; (D) the present form of *C. japonica*. The number (IV) in the figure indicate the formed repetitive sequence (see Figure 10 and additional file 3), and its transition position during genome rearrangement.

We described above the relationship between the *clp*P pseudogene, within the *trn*N-GUU gene to *chl*L gene segment, and genome rearrangements. In the *Adonis annua *cp genome [37], the functions of the *clp*P gene are thought to have been lost as a result of genome rearrangement (inversion event). In the *pet*A to *clp*P region of the *C. japonica *cp genome, assuming a *C. taitungensis*-like ancestral cp genome, we can construct a genome rearrangement model in which a minimum of three inversions would be required to transform the gene order of the *C. taitungensis *cp genome into that of *C. japonica *(Figure 11). The *clp*P pseudogene in the *C. japonica *cp genome was apparently caused by such genome rearrangements, and the repetitive sequences halfway between *psb*J and *clp*P, and between *ccs*A and *pet*A in the *C. japonica *cp genome should therefore be vestiges of the genome rearrangements.

## Conclusion

This study has revealed that the coniferous species, *C. japonica*, has a distinct cp genome compared to previously reported land plant cp genomes. In terms of gene content, several genes in the *C. japonica *cp genome differ significantly, having either been lost or diverged, from those of other land plants, while the gene order and genome structure also differ significantly. The deleted large IRs and the numerous genome rearrangements that have occurred in the *C. japonica *cp genome have provided new insights into the evolutionary lineage of conifers. However, as the complete cp genome nucleotide sequences of only three conifer species that belong to two distinct genera have been determined, our present results will certainly advance our understanding of the complex evolutionary history of the coniferous cp genome.

## Methods

### Isolation of chloroplast DNA

Open-pollinated *C. japonica *seeds were collected from several clones, and were germinated and grown for 1 month in a greenhouse. *C. japonica *chloroplasts were isolated from the needle tissues of these seedlings using the sucrose density gradient method [63]. The chloroplast pellet was resuspended in 250 ml of Kool's buffer A (50 mM Tris-HCl, pH 8.0, 0.35 M sucrose, 7 mM EDTA, 5 mM 2-mercaptoethanol) containing 0.1% bovine serum albumin, and the suspension was filtered through layers of cheesecloth and Miracloth (Calbiochem; without squeezing). The filtrate was centrifuged, and the resulting green pellet was resuspended in 2.5 ml of Kool's buffer A. This second suspension was then loaded onto a stepwise 20–45–55% sucrose gradient in 50 mM Tris-HCl, pH 8.0, 0.3 M sorbitol, 7 mM EDTA, and centrifuged for 30 min. The green band at the 20–45% sucrose interphase was collected, diluted 1:3 with Kool's buffer B (50 mM Tris-HCl, pH 8.0, 20 mM EDTA), centrifuged for 10 min, and the chloroplast pellet then resuspended in Kool's buffer B. The chloroplasts were lysed by adding SDS to a final concentration of 3%. A 1/20th volume of 10 mg/ml pronase E was added to the solution, and the mixture incubated overnight at 37°C. DNA was extracted twice from the lysate with phenol and once with phenol/chloroform/isoamyl alcohol (25:24:1), and the DNA was precipitated with 0.1 volumes of 3 M sodium acetate and 2.5 volumes of ethanol. The precipitate was washed twice with 70% ethanol and dissolved in water. The extracted DNAs were further purified using the DNeasy Plant Mini Kit (QIAGEN) and treated with ATP-dependent DNase (TOYOBO) to remove linear double- or single-stranded DNA.

### Chloroplast DNA sequencing and genome assembly

The cp DNA isolated was sheared by ultrasonication, and the sheared fragments then blunted and cloned into pBluescript II vector. The cp DNA fragments were shotgun sequenced using the BigDye Terminator Cycle Sequencing v3.1™ Kit with an ABI 3100 Genetic Analyzer (both PE Applied Biosystems). Sequencher 3.1 (Gene Codes Corporation) software was used for sequence analysis and assembly. The sonication-derived cloned fragments were found to cover 80% of the whole genome after contig assembly. Any remaining sequence gaps were amplified by PCR and sequenced directly from the amplification products.

### Gene annotation

The cp genome of *C. Japonica *was annotated using DOGMA [Dual Organellar GenoMe Annotator, 64] after a FASTA-formatted file of the complete cp genome was uploaded to the program's server. Gene annotation and comparative genome analyses (BLASTN, BLASTX) were performed against a custom database of 11 previously published cp genomes using default parameters of 60% for protein coding genes and 85% for tRNAs and rRNAs. For genes with low amino acid sequence identity, manual annotation was performed using a percentage identity threshold of 25–50%. The fully annotated cp genome of *Cryptomeria japonica *was submitted to DDBJ GenBank with the following accession number [DDBJ: AP009377].

### Exploration of the differences in gene contents and diversified genes

Exploration of the differences in gene contents and diversified genes between the *C. japonica *cp genome and the six previously published cp genomes was performed using PipMaker [65]. The six cp genomes compared are as follows: the dicot angiosperm, *E. globulus *(*Myrtaceae*, 160,286 bp, AY780259); the monocot angiosperm, *O. sativa *(*Poaceae*, 134,525 bp, X15901); the liverwort, *M. polymorpha *(*Marchantiaceae*, 121,024 bp, NC001319); the fern, *A. capillus *(*Pteridaceae*, 150,568 bp, AY178864); and the two gymnosperms, *C. taitungensis *(*Cycadaceae*, 163,403 bp, AP009339) and *P. thunbergii *(*Pinaceae*, 119,707 bp, D17510). The variable genes identified within the *C. japonica *cp genome by gene annotations were aligned with the corresponding coding genes of the six land plant cp genomes using ClustalX [66] followed by screening for nucleotide and amino acid sequence differences.

### Comparative analysis of genome structure

Comparative analysis of the genome structure of the seven cp genomes, including that of the *C. japonica *cp genome, was performed using the Harr-plot analysis of PipMaker [65]. For estimates of genome rearrangement, the GRIMM web server [67] was used to identify the minimum number of rearrangements by inversion in pairwise comparisons of the cp genome. GRIMM cannot deal with duplicated genes and requires that the genomes that are compared have the same gene content, so that one of the two IR copies and their genes were arbitrarily excluded.

### Examination of dispersed repeat sequences

FASTPCR software [68] was used to locate and count the direct (forward) and inverted (palindromic) repeats within the *C. japonica *cp genome. The identification of repeat sequences was assessed with the following parameters: options at a minimum length of 50 bp and 90% or greater sequence identity.

### Phylogenetic analysis using the rbcL gene of chloroplast genome

Based on the *rbc*L gene sequence of the *C. japonica *cp genome, the *rbc*L gene nucleotide sequences of 132 gymnosperm species and eight out-group species were obtained by a FASTA search of GenBank. The DNA sequences were aligned using ClustalX [66], with excluded gap regions. Phylogenetic analysis using the neighbor-joining (NJ) method was performed using ClustalW from the DDBJ web server [69]. The Kimura-2-parameter model of molecular evolution was used in the NJ method of the nucleotide sequences. Bootstrap analysis was performed for the NJ method with 100 replicates.

## Abbreviations

cp genome: chloroplast genome; IR: inverted repeat; SSC: small single copy; LSC: large single copy; bp: base pair; ycf: hypothetical chloroplast reading frame; IGS: intergenic spacer.

## Authors' contributions

TH completed the *C. japonica *cp genome sequence, performed the annotations, conducted the comparative analyses, prepared the DDBJ GenBank submissions, and drafted the manuscript; AW conceived of the project, sequenced the greater part of the *C. japonica *cp genome, and drafted the manuscript; MK assisted in the preparation of the sequencing templates and helped with the annotations; TK contributed to the design of the project. KT conceived of the project and drafted the manuscript. All authors assisted with manuscript preparation and read and approved the final draft.

## Supplementary Material

Additional file 1 **Harr plot analyses comparing the cp genome of *C. taitungensis *with those of *E. globulus *and *P. thunbergii***. Each dotplot shows the positions where 45 out of 50 nucleotides match in the two sequences. The plot analysis was carried out using Pipmaker software. Sequences along the Y-axis are set from the top to the bottom, and along the X-axis are from left to right. Relative lengths of sequences are shown to the side and below the boxes. The colored gene segments along the X- and Y-axes correspond with common gene units of the seven cp genomes (shown in Figure 7). At the expected endpoint of inversion or translocation mutation, the gene name is attached based on the X-axis cp genome. The pseudogene is indicated by ψ (pseudo-). Click here for file

Additional file 2 **The neighbor-joining tree of the *rbc*L gene in gymnosperms**. The branch length indicates the number of substitutions. The numbers at each node denote the traditional bootstrap replicates that support the monophyly of the taxa in the subset designated by the node. Only bootstrap values higher than 50% are shown. The species highlighted in red represent the cp genomes of gymnosperms already determined. Click here for file

Additional file 3 **The character of dispersed repetitive sequences at expected inversion or translocation endpoints**. The character of each repetitive sequence is indicated by similarity, length, repeat type, location, and sequence. The positions of each repetitive sequence correspond with the numbers (I-IV) above the gene segments of the *C. japonica *cp genome (see Figure 10). The bold characters indicate the location of repeat sequences, and IGS indicates the intergenic spacer region. Click here for file

## References

[B1] Shinozaki K, Ohme M, Tanaka M, Wakasugi T, Hayashida N, Matsubayashi T, Zaita N, Chunwongse J, Obokata J, Yamaguchi-Shinozaki K, Ohto C, Torazawa K, Meng BY, Sugita M, Deno H, Kamogashira T, Yamada K, Kusuda J, Takaiwa F, Kato A, Tohdoh N, Shimada H, Sugiura M (1986). The complete nucleotide sequence of the tobacco chloroplast genome: its gene organization and expression. EMBO J.

[B2] Ohyama K, Fukuzawa H, Kohchi T, Shirai H, Sano T, Sano S, Umesono K, Shiki Y, Takeuchi M, Chang Z, Aota S, Inokuchi H, Ozeki H (1986). Chloroplast gene organization deduced from complete sequence of liverwort *Marchantia polymorpha *chloroplast DNA. Nature.

[B3] Jansen RK, Kaittanis C, Saski C, Lee SB, Tomkins J, Alverson AJ, Daniell H (2006). Phylogenetic analysis of Vitis (Vitaceae) based on complete chloroplast genome sequences: effects of taxon sampling and phylogenetic methods on resolving relationships among rosids. BMC Evol Biol.

[B4] Lee SB, Kaittanis C, Jansen RK, Hostetler JB, Tallon LJ, Town CD, Daniell H (2006). The complete chloroplast genome sequence of *Gossypium hirsutum *: organization and phylogenetic relationships to other angiosperms. BMC Genomics.

[B5] Bausher MG, Singh ND, Lee SB, Jansen RK, Daniell H (2006). The complete chloroplast genome sequence of *Citrus sinensis *(L.) Osbeck var 'Ridge Pineapple': organization and phylogenetic relationships to other angiosperms. BMC Plant Biol.

[B6] Cai Z, Penaflor C, Kuehl JV, Leebens-Mack J, Carlson JE, dePamphilis CW, Boore JL, Jansen RK (2006). Complete plastid genome sequences of Drimys, Liriodendron, and Piper: implications for the phylogenetic relationships of magnoliids. BMC Evol Biol.

[B7] Ruhlman T, Lee SB, Jansen RK, Hostetler JB, Tallon LJ, Town CD, Daniell H (2006). Complete plastid genome sequence of Daucus carota: Implications for biotechnology and phylogeny of angiosperms. BMC Genomics.

[B8] Wu CS, Wang YN, Liu SM, Chaw SM (2007). Chloroplast Genome (cpDNA) of *Cycas taitungensis *and 56 cp Protein-Coding Genes of *Gnetum parvifolium*: Insights into cp DNA Evolution and Phylogeny of Extant Seed Plants. Mol Biol Evol.

[B9] Wakasugi T, Tsudzuki J, Ito S, Nakashima K, Tsudzuki T, Sugiura M (1994). Loss of all ndh genes as determined by sequencing the entire chloroplast genome of the black pine *Pinus thunbergii*. Proc Natl Acad Sci USA.

[B10] Noh EW, Lee JS, Choi YI, Han MS, Yi YS, Han SU Complete nucleotide sequence of *Pinus koraiensis*.

[B11] Neale DB, Sederoff RR (1989). Paternal inheritance of chloroplast DNA and maternal inheritance of mitochondrial DNA in loblolly pine. Theor Appl Genet.

[B12] Szmidt AE, Alden T, Hallgren JE (1987). Paternal inheritance of chloroplast DNA in Larix. Plant Mol Biol.

[B13] Szmidt AE, El-Kassaby YA, Sigurgeirsson A, Alden T, Lindgren D, Hallgren JE (1988). Classifying seedlots of *Picea sitchensis *and *P. glauca *in zones of introgression using restriction analysis of chloroplast DNA. Theor Appl Genet.

[B14] Neale DB, Marshall KA, Sederoff RR (1989). Chloroplast and mitochondrial DNA are paternally inherited in *Sequoia sempervirens *D.Don Endl. Proc Natl Acad Sci USA.

[B15] Kondo T, Tsumura Y, Kawahara T, Okamura M (1998). Paternal inheritance of chloroplast and mitochondrial DNA in interspecific hybrids of *Chamaecyparis *spp. Breed Sci.

[B16] Seido K, Maeda H, Shiraishi S (2000). Determination of the selfing rate in a Hinoki (*Chamaecyparis obtsusa*) seed orchard by using a chloroplast PCR-SSCP marker. Silvae Genetica.

[B17] Chen J, Tauer C, Huang Y (2002). Paternal chloroplast inheritance patterns in pine hybrids detected with *trn *L-*trn*F intergenic region polymorphism. Theor Appl Genet.

[B18] Wagner DB, Furnier GR, Saghai-Maroof MA, Williams SM, Danick BP, Allard RW (1987). Chloroplast DNA polymorphisms in lodgepole and jack pines and their hybrids. Proc Natl Acad Sci USA.

[B19] Hong YP, Hipkins VD, Strauss SH (1993). Chloroplast DNA Diversity Among Trees, Populations and Species in the California Closed-Cone Pines (*Pinus radiate*, *Pinus muricata *and *Pinus attenuate*). Genetics.

[B20] Dong J, Wagner DB (1994). Paternally Inherited Chloroplast Polymorphism in Pinus: Estimation of Diversity and Population Subdivision, and Tests of Disequilibrium With a Maternally Inherited Mitochondrial Polymorphism. Genetics.

[B21] Tsumura Y, Suyama Y, Taguchi H, Ohba K (1994). Geographical cline of chloroplast DNA variation in *Abies mariesii*. Theor Appl Genet.

[B22] Wakasugi T, Hirose T, Horihata M, Tsudzuki T, Kosselw H, Sugiura M (1996). Creation of a novel protein-coding region at the RNA level in black pine chloroplasts: The pattern of RNA editing in the gymnosperm chloroplast is different from that in angiosperms. Proc Natl Acad Sci USA.

[B23] Sugiura M (1989). The chloroplast chromosomes in land plants. Annu Rev Cell Biol.

[B24] Sugiura M (1992). The chloroplast genome. Plant Mol Biol.

[B25] Lidholm J, Szmidt AE, Hallgren JE, Gustafsson P (1988). The chloroplast genomes of conifers lack one of the rRNA-encoding inverted repeats. Mol Gen Genet.

[B26] Strauss SH, Palmer JD, Howe GT, Doersken AH (1988). Chloroplast genomes of two conifers lack a large inverted repeat and are extensively rearranged. Proc Natl Acad Sci USA.

[B27] Tsumura Y, Ogihara Y, Sasakuma T, Ohba K (1993). Physical map of chloroplast DNA in sugi, *Cryptomeria japonica*. Theor Appl Genet.

[B28] Palmer JD, Stein DB (1986). Conservation of chloroplast genome structure among vascular plants. Curr Genet.

[B29] Tsudzuki J, Nakashima K, Tsudzuki T, Hiratsuka J, Shibata M, Wakasugi T, Sugiura M (1992). Chloroplast DNA of black pine retains a residual inverted repeat lacking rRNA genes: nucleotide sequences of *trnQ*, *trnK*, *psbA*, *trnI *and *trnH *and the absence of *rps16*. Mol Gen Genet.

[B30] White EE (1990). Chloroplast DNA in *Pinus monticola*. 1. Physical map. Theor Appl Genet.

[B31] Lidholm J, Gustafsson P (1991). The chloroplast genome of the gymnosperm *Pinus contorta *: a physical map and a complete collection of overlapping clones. Curr Genet.

[B32] Steane DA (2005). Complete Nucleotide Sequence of the Chloroplast Genome from the Tasmania Blue Gum, *Eucalyptus globules *(Myrtaceae). DNA Res.

[B33] Chaw SM, Zharkikh A, Sung HM, Lau TC, Li WH (1997). Molecular phylogeny of extant gymnosperms and seed plant evolution: analysis of nuclear 18s rRNA sequence. Mol Biol Evol.

[B34] Chaw SM, Parkinson CL, Cheng Y, Vincent T, Palmer JD (2000). Seed plant phylogeny inferred from all three plant genomes: Monophyly of extant gymnosperms and origin of Gnetales from conifers. Proc Natl Acad Sci USA.

[B35] Shimada H, Sugiura M (1991). Fine structural features of the chloroplast genome: comparison of the sequenced chloroplast genomes. Nucleic Acids Res.

[B36] Umesono K, Inokuchi H, Shiki Y, Takeuchi M, Chang Z, Fukuzawa H, Kohchi T, Shirai H, Ohyama K, Ozeki H (1988). Structure and organization of *Marchantia polymorpha *chloroplast genome II. Gene organization of the large single copy region from *rps*12 to *atp*B. J Mol Biol.

[B37] Downie SR, Palmer JD, Soltis PS, Soltis DE, Doyle JJ (1992). Use of chloroplast DNA rearrangements in reconstructing plant phylogeny. Molecular systematic of plants.

[B38] Doyle JJ, Doyle JL, Palmer JD (1995). Multiple independent losses of two genes and one intron from legume chloroplast genomes. Syst Bot.

[B39] Johansson JT (1999). There large inversions in the chloroplast genomes and one loss of the chloroplast gene *rps *16 suggest an early evolutionary split in the genus *Adonis *(Ranunculaceae). Plant Syst Evol.

[B40] Saski C, Lee SB, Daniell H, Wood TC, Tomkins J, Kim HG, Jansen RK (2005). Complete chloroplast genome sequence of *Glycin max *and comparative analyses with other legume genomes. Plant Mol Biol.

[B41] Tsuji S, Ueda K, Nishiyama T, Hasebe M, Yoshikawa S, Konagaya A, Nishiuchi T, Yamaguchi K (2007). The chloroplast genome from a lycophyte (microphyllophyte), *Selaginella uncinata*, has a unique inversion, transpositions and many gene losses. J Plant Res.

[B42] Kugita M, Kaneko A, Yamamoto Y, Takeya Y, Matsumoto T, Yoshinaga K (2003). The complete nucleotide sequence of the hornwort (*Anthoceros formosae*) chloroplast genome: insight into the earliest land plants. Nucleic Acids Res.

[B43] Sugiura C, Sugita M (2004). Plastid transformation reveals that moss tRNA^Arg^-CCG is not essential for plastid function. The Plant J.

[B44] Chumley TW, Palmer JD, Mower JP, Fourcade HM, Calie PJ, Boore JL, Jansen RK (2006). The complete chloroplast genome sequence of Pelargonium × hortorum: Organization and evolution of the largest and most highly rearranged chloroplast genome of land plants. Mol Biol Evol.

[B45] Maier RM, Neckermann K, Igloi GL, Kossel H (1995). Complete Sequence of the Maize Chloroplast Genome: Gene Content, Hotspots of Divergence and Fine Tuning of Genetic Information by Transcript Editing. J Mol Biol.

[B46] Kohchi T, Ogura Y, Umesono K, Yamada Y, Komano T, Ohyama K (1988). Ordered processing and splicing in a polycistronic transcript in liverwort chloroplasts. Curr Genet.

[B47] Clarke AK, Gustafsson P, Lidholm JÅ (1994). Identification and expression of the chloroplast *clp *P gene in the conifer *Pinus contorta*. Plant Mol Biol.

[B48] Kanno A, Hirai A (1993). A transcription map of the chloroplast genome from rice (*Oryza sativa*). Curr Genet.

[B49] Boudreau E, Takahashi Y, Lemieux C, Turmel M, Rochaix JD (1997). The chloroplast *ycf3 *and *ycf4 *open reading frames of *Chlamydomonas reinhardtii *are required for the accumulation of the photosystem l complex. The EMBO J.

[B50] Drescher A, Ruf S, Calsa T, Carrer H, Bock R (2000). The two largest chloroplast genome-encoded open reading frames of higher plants are essential genes. Plant J.

[B51] Hiratsuka J, Shimada H, Whittier R, Ishibashi T, Sakamoto M, Mori M, Kondo C, Honji Y, Sun CR, Meng BY, Li YQ, Kanno A, Nishizawa Y, Hirai A, Shinozaki K, Sugiura M (1989). The complete sequence of the rice (*Oryza sativa*) chloroplast genome: intermolecular recombination between distinct tRNA genes accounts for a major plastid DNA inversion during the evolution of cereals. Mol Gen Genet.

[B52] Raubenson LA, Peery R, Chumley TW, Dziubek C, Fourcade HM, Boore JL, Jansen RK (2007). Comparative chloroplast genomics: analyses including new sequences from the angiosperms *Nuphar advena *and *Ranunculus macranthus*. BMC genomics.

[B53] Palmer JD, Thompson WF (1981). Rearrangements in the chloroplast genomes of mung bean and pea. Proc Natl Acad Sci USA.

[B54] Lavin M, Doyle JJ, Palmer JD (1990). Evolutionary significance of the loss of the chloroplast-DNA inverted repeat in the Leguminosae subfamily Papilionoidae. Evolution.

[B55] Liston A, Crisp M, Doyle J (1995). Use of the polymerase chain reaction to survey for the loss of the inverted repeat in the legume chloroplast genome. Advances in legume systematics Phylogeny.

[B56] Palmer JD, Thompson WF (1982). Chloroplast DNA rearrangements are more frequent when a large inverted repeat sequence is lost. Cell.

[B57] Cosner ME, Raubenson LA, Jansen RK (2004). Chloroplast DNA rearrangements in Campanulaceae: phylogenetic utility of highly rearranged genomes. BMC Evol Biol.

[B58] Tsai CH, Strauss SH (1989). Dispersed repetitive sequences in the chloroplast genome of Douglas-fir. Curr Genet.

[B59] Hipkins VD, Marshall KA, Neale DB, Rottmann WH, Strauss SH (1995). A mutation hotspot in the chloroplast genome of a conifer (Douglas-fir: *Pseudotsuga*) is caused by variability in the number of direct repeats derived from a partiall duplicated tRNA gene. Curr Genet.

[B60] Quigley F, Weil JH (1985). Organization and sequence of five tRNA genes and of an unidentified reading frame in the wheat chloroplast genome: evidence for gene rearrangements during the evolution of chloroplast genomes. Curr Genet.

[B61] Howe CJ (1985). The endpoints of an inversion in wheat chloroplast DNA are associated with short repeated sequences containing homology to *att*-lamba. Curr Genet.

[B62] Shimada H, Sugiura M (1989). Pseudogenes and short repeated sequences in the rice chloroplast genome. Curr Genet.

[B63] Ogihara Y, Tsunewaki K (1982). Molecular basis of the genetic diversity of the cytoplasm in *Triticum *and *Aegilops*. Diversity of chloroplast genome and its lineage revealed by the restriction pattern of ct-DNAs. Jpn J Genet.

[B64] Wyman SK, Jansen RK, Boore JL (2004). Automatic annotation of organellar genomes with DOGMA. Bioinformatics.

[B65] Schwartz S, Elnitski L, Li M, Weirauch M, Riemer C, Smit A, Program NCS, Green ED, Hardison RC, Miller W (2003). MultiPipMaker and supporting tools: Alignments and analysis of multiple genomic DNA sequences. Nucleic Acids Res.

[B66] Higgins DG, Thompson JD, Gibson TJ (1996). Using CLUSTAL for multiple sequence aligments. Methods Enzymol.

[B67] Tesler G (2002). GRIMM: genome rearrangements web server. Bioinformatics.

[B68] Kalendar R (2005). *FASTPCR *– PCR primer design, DNA and protein tool, repeats and own database searches program. http://www.biocenter.Helsinki.fi/bi/Programs/fastpcr.htm.

[B69] DNA Data Bank of Japan. http://www.ddbj.nig.ac.jp/index-j.html.

