# Supplementary material for: Complete nucleotide sequence of the Cryptomeria japonica D. Don. chloroplast genome and comparative chloroplast genomics: diversified genomic structure of coniferous species
Source: BMC Plant Biol. 2008 Jun 23;8:70. doi: 10.1186/1471-2229-8-70 (PMC2443145; doi:10.1186/1471-2229-8-70)
Supplement: Additional file 1 — Harr plot analyses comparing the cp genome of C. taitungensis with those of E. globulus and P. thunbergii. Each dotplot shows the positions where 45 out of 50 nucleotides match in the two sequences. The plot analysis was carried out using Pipmaker software. Sequences along the Y-axis are set from the top to the bottom, and along the X-axis are from left to right. Relative lengths of sequences are shown to the side and below the boxes. The colored gene segments along the X- and Y-axes correspond with common gene units of the seven cp genomes (shown in Figure 7). At the expected endpoint of inversion or translocation mutation, the gene name is attached based on the X-axis cp genome. The pseudogene is indicated by ψ (pseudo-). [file 1471-2229-8-70-S1.pdf]

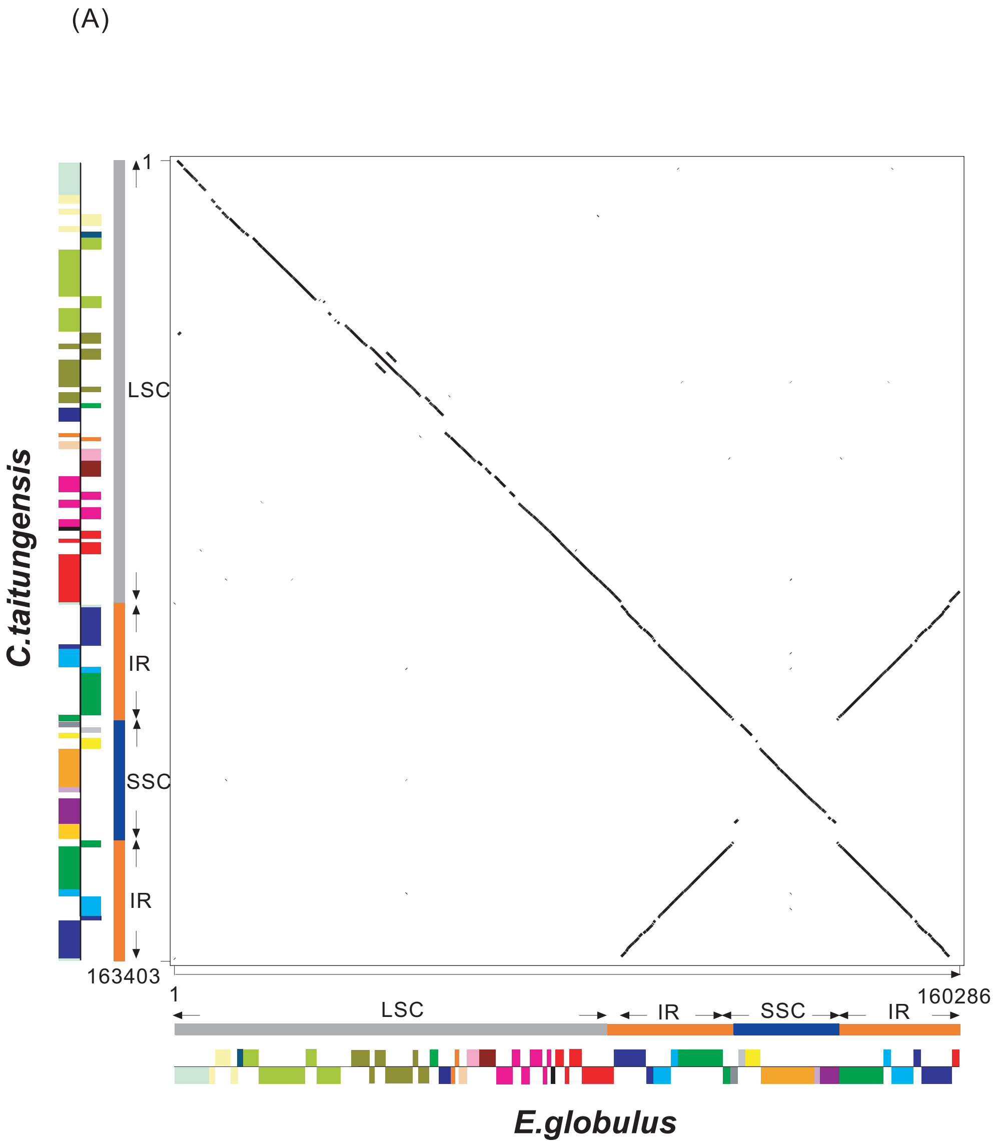

(A)
C.taitungensis
1
LSC
IR
SSC
IR
163403
1
160286
LSC
IR
SSC
IR
E.globulus

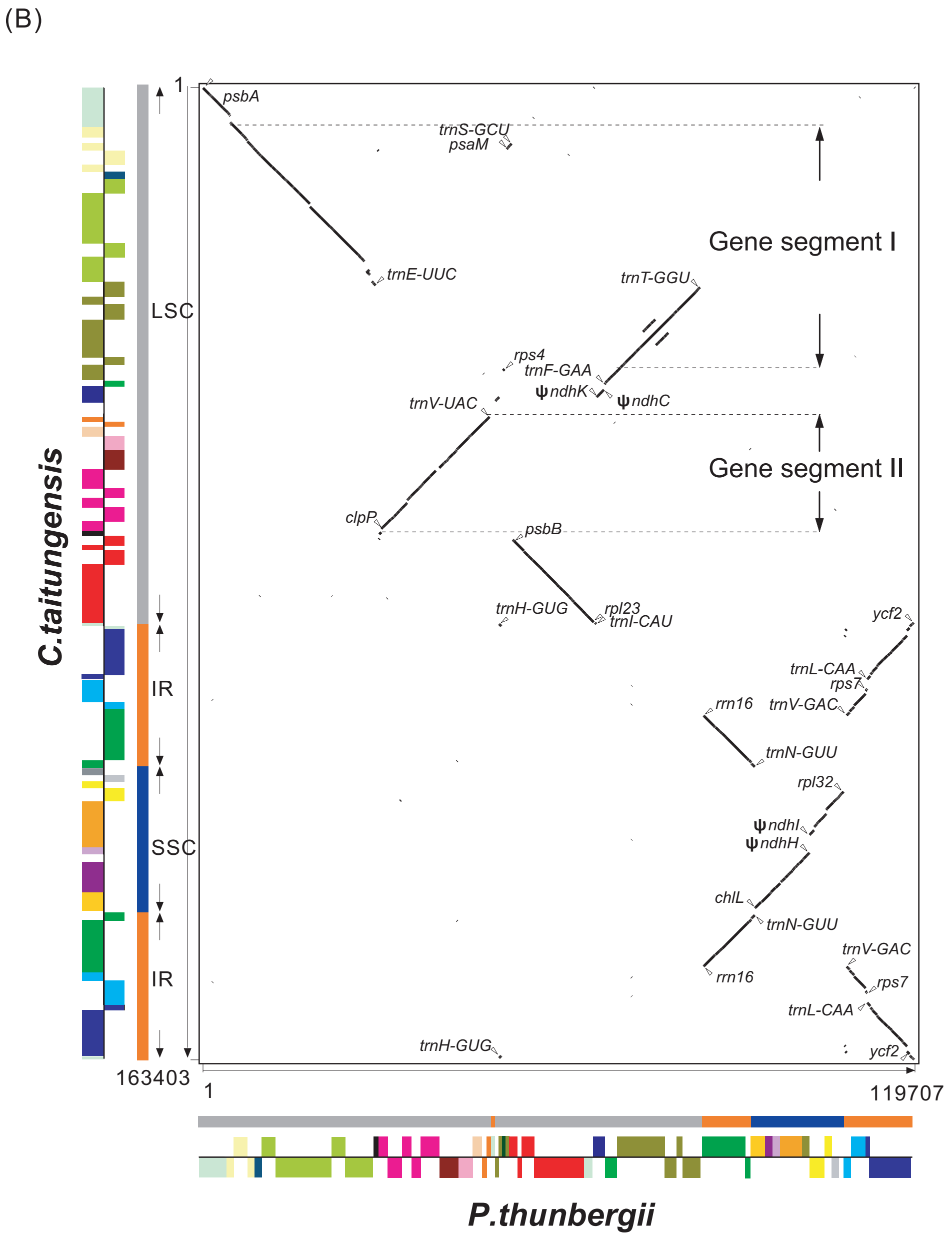

(B)
C.taitungensis
1
LSC
IR
SSC
IR
163403
psbA
trnS-GCU
psaM
Gene segment I
trnE-UUC
trnT-GGU
rps4
trnF-GAA
ψndhK
ψndhC
trnV-UAC
Gene segment II
clpP
psbB
trnH-GUG
rpl23
trnI-CAU
ycf2
trnL-CAA
rps7
trnV-GAC
rrn16
trnN-GUU
rpl32
ψndhI
ψndhH
chlL
trnN-GUU
trnV-GAC
rrn16
rps7
trnL-CAA
trnH-GUG
ycf2
1
119707
P.thunbergii
