# Supplementary material for: Complete nucleotide sequence of the Cryptomeria japonica D. Don. chloroplast genome and comparative chloroplast genomics: diversified genomic structure of coniferous species
Source: BMC Plant Biol. 2008 Jun 23;8:70. doi: 10.1186/1471-2229-8-70 (PMC2443145; doi:10.1186/1471-2229-8-70)
Supplement: Additional file 2 — The neighbor-joining tree of the rbcL gene in gymnosperms. The branch length indicates the number of substitutions. The numbers at each node denote the traditional bootstrap replicates that support the monophyly of the taxa in the subset designated by the node. Only bootstrap values higher than 50% are shown. The species highlighted in red represent the cp genomes of gymnosperms already determined. [file 1471-2229-8-70-S2.pdf]

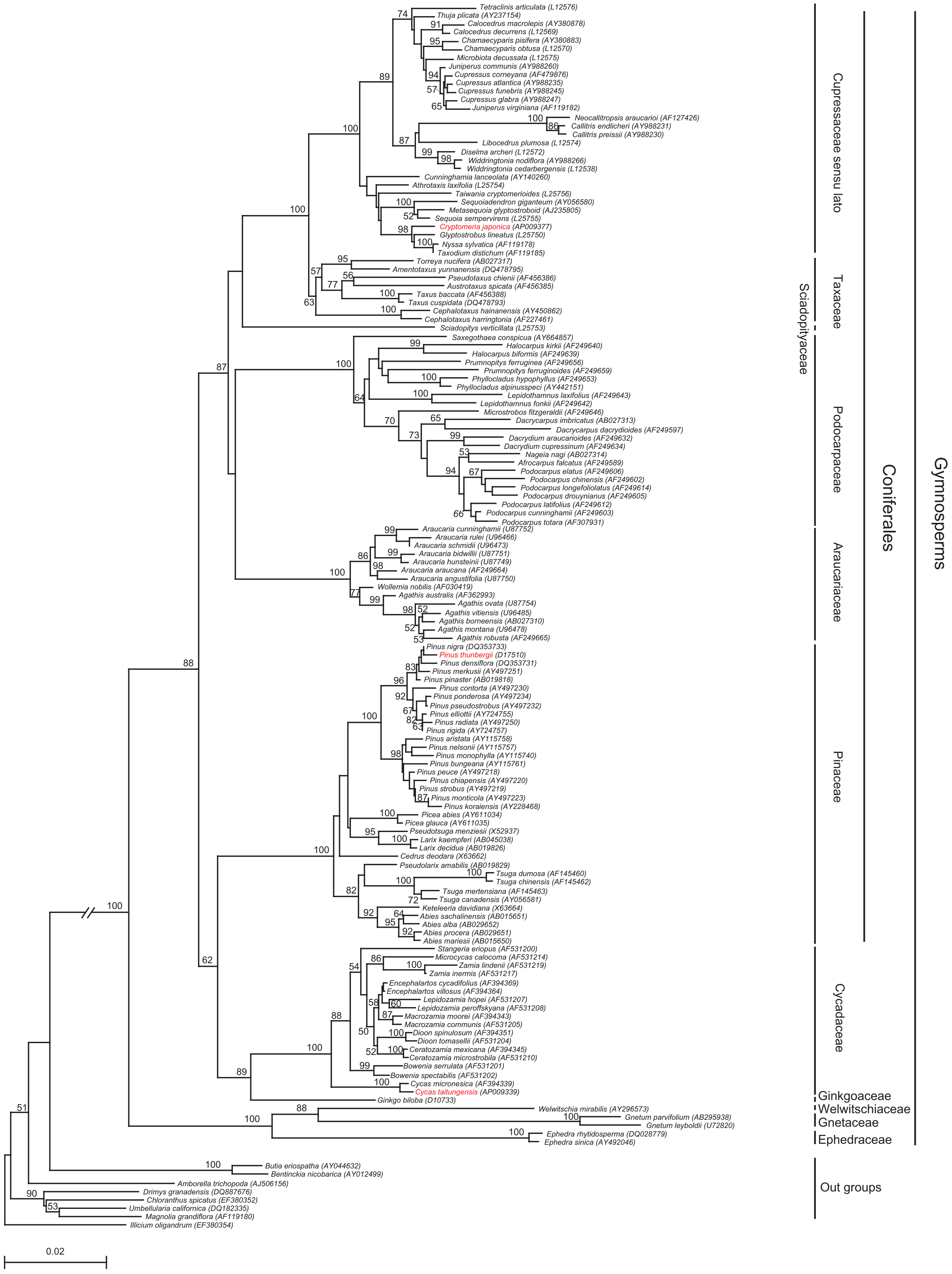

Tetraclinis articulata (L12576)
Thuja plicata (AY237154)
Calocedrus macrolepis (AY380878)
Calocedrus decurrens (L12569)
Chamaecyparis pisifera (AY380883)
Chamaecyparis obtusa (L12570)
Microbiota decussata (L12575)
Juniperus communis (AY988260)
Cupressus corneyana (AF479876)
Cupressus atlantica (AY988235)
Cupressus funebris (AY988245)
Cupressus glabra (AY988247)
Juniperus virginiana (AF119182)
Neocallitropsis araucarioi (AF127426)
Callitris endlicheri (AY988231)
Callitris preissii (AY988230)
Libocedrus plumosa (L12574)
Diselma archeri (L12572)
Widdringtonia nodiflora (AY988266)
Widdringtonia cedarbergensis (L12538)
Cunninghamia lanceolata (AY140260)
Athrotaxis laxifolia (L25754)
Taiwania cryptomerioides (L25756)
Sequoiadendron giganteum (AY056580)
Metasequoia glyptostroboid (AJ235805)
Sequoia sempervirens (L25755)
Cryptomeria japonica (AP009377)
Glyptostrobus lineatus (L25750)
Nyssa sylvatica (AF119178)
Taxodium distichum (AF119185)
Torreya nucifera (AB027317)
Amentotaxus yunnanensis (DQ478795)
Pseudotaxus chienii (AF456386)
Austrotaxus spicata (AF456385)
Taxus baccata (AF456388)
Taxus cuspidata (DQ478793)
Cephalotaxus hainanensis (AY450862)
Cephalotaxus harringtonia (AF227461)
Sciadopitys verticillata (L25753)
Saxegothaea conspicua (AY664857)
Halocarpus kirkii (AF249640)
Halocarpus biformis (AF249639)
Prumnopitys ferruginea (AF249656)
Prumnopitys ferruginoides (AF249659)
Phyllocladus hypophyllus (AF249653)
Phyllocladus alpinusspeci (AY442151)
Lepidothamnus laxifolius (AF249643)
Lepidothamnus fonkii (AF249642)
Microstrobos fitzgeraldii (AF249646)
Dacrycarpus imbricatus (AB027313)
Dacrycarpus dacrydioides (AF249597)
Dacrydium araucarioides (AF249632)
Dacrydium cupressinum (AF249634)
Nageia nagi (AB027314)
Afrocarpus falcatus (AF249589)
Podocarpus elatus (AF249606)
Podocarpus chinensis (AF249602)
Podocarpus longefoliolatus (AF249614)
Podocarpus drouynianus (AF249605)
Podocarpus latifolius (AF249612)
Podocarpus cunninghamii (AF249603)
Podocarpus totara (AF307931)
Araucaria cunninghamii (U87752)
Araucaria rulei (U96466)
Araucaria schmidii (U96473)
Araucaria bidwillii (U87751)
Araucaria hunsteinii (U87749)
Araucaria araucana (AF249664)
Araucaria angustifolia (U87750)
Wollemia nobilis (AF030419)
Agathis australis (AF362993)
Agathis ovata (U87754)
Agathis vitiensis (U96485)
Agathis borneensis (AB027310)
Agathis montana (U96478)
Agathis robusta (AF249665)
Pinus nigra (DQ353733)
Pinus thunbergii (D17510)
Pinus densiflora (DQ353731)
Pinus merkusii (AY497251)
Pinus pinaster (AB019818)
Pinus contorta (AY497230)
Pinus ponderosa (AY497234)
Pinus pseudostrobus (AY497232)
Pinus elliottii (AY724755)
Pinus radiata (AY497250)
Pinus rigida (AY724757)
Pinus aristata (AY115758)
Pinus nelsonii (AY115757)
Pinus monophylla (AY115740)
Pinus bungeana (AY115761)
Pinus peuce (AY497218)
Pinus chiapensis (AY497220)
Pinus strobus (AY497219)
Pinus monticola (AY497223)
Pinus koraiensis (AY228468)
Picea abies (AY611034)
Picea glauca (AY611035)
Pseudotsuga menziesii (X52937)
Larix kaempferi (AB045038)
Larix decidua (AB019826)
Cedrus deodara (X63662)
Pseudolarix amabilis (AB019829)
Tsuga dumosa (AF145460)
Tsuga chinensis (AF145462)
Tsuga mertensiana (AF145463)
Tsuga canadensis (AY056581)
Keteleeria davidiana (X63664)
Abies sachalinensis (AB015651)
Abies alba (AB029652)
Abies procera (AB029651)
Abies mariesii (AB015650)
Stangeria eriopus (AF531200)
Microcycas calocoma (AF531214)
Zamia lindenii (AF531219)
Zamia inermis (AF531217)
Encephalartos cycadifolius (AF394369)
Encephalartos villosus (AF394364)
Lepidozamia hopei (AF531207)
Lepidozamia peroffskyana (AF531208)
Macrozamia moorei (AF394343)
Macrozamia communis (AF531205)
Dioon spinulosum (AF394351)
Dioon tomasellii (AF531204)
Ceratozamia mexicana (AF394345)
Ceratozamia microstrobila (AF531210)
Bowenia serrulata (AF531201)
Bowenia spectabilis (AF531202)
Cycas micronesica (AF394339)
Cycas taitungensis (AP009339)
Ginkgo biloba (D10733)
Welwitschia mirabilis (AY296573)
Gnetum parvifolium (AB295938)
Gnetum leyboldii (U72820)
Ephedra rhytidosperma (DQ028779)
Ephedra sinica (AY492046)
Butia eriospatha (AY044632)
Bentinckia nicobarica (AY012499)
Amborella trichopoda (AJ506156)
Drimys granadensis (DQ887676)
Chloranthus spicatus (EF380352)
Umbellularia californica (DQ182335)
Magnolia grandiflora (AF119180)
Illicium oligandrum (EF380354)
0.02
Cupressaceae sensu lato
Taxaceae
Sciadopityaceae
Podocarpaceae
Araucariaceae
Pinaceae
Cycadaceae
Ginkgoaceae
Welwitschiaceae
Gnetaceae
Ephedraceae
Out groups
Coniferales
Gymnosperms
