# Supplementary material for: Complete nucleotide sequence of the Cryptomeria japonica D. Don. chloroplast genome and comparative chloroplast genomics: diversified genomic structure of coniferous species
Source: BMC Plant Biol. 2008 Jun 23;8:70. doi: 10.1186/1471-2229-8-70 (PMC2443145; doi:10.1186/1471-2229-8-70)
Supplement: Additional file 3 — The character of dispersed repetitive sequences at expected inversion or translocation endpoints. The character of each repetitive sequence is indicated by similarity, length, repeat type, location, and sequence. The positions of each repetitive sequence correspond with the numbers (I-IV) above the gene segments of the C. japonica cp genome (see Figure 10). The bold characters indicate the location of repeat sequences, and IGS indicates the intergenic spacer region. [file 1471-2229-8-70-S3.pdf]

(I) Similarity : 98%
Length : 61 bp
Repeat type : Direct-Antisense reverse repeat
Location : *clpP*-**IGS**-*accD* (from 25205 to 25145)
trnL-CAA-**IGS**-*ycf1* (from 121996 to 122056)

```
 25205   : AAATGTGCCG AAAGTGCCCT ATCAAGGGCC CGAAGAGGAG GAAGCAAACT GGGTCGAATT A  25145
         : |||||||||| |||||||| | |||||||||| |||||||||| |||||||:|| |||||||||| |
121996   : AAATGTGCCG AAAGTGCCAT ATCAAGGGCC CGAAGAGGAG GAAGCAAGCT GGGTCGAATT A  122056
```

(II) Similarity : 93%
Length : 55 bp
Repeat type : Direct-Antisense reverse repeat
Location : *rbcL*-**IGS**-*atpE* (from 30258 to 30204)
trnL-CAA-**IGS**-*ycf1* (from 122082 to 122136)

```
 30258   : CTTTACGTTC GACACCCAGG TCGAGTAGAC CTCGTTCTTG TAAGAGCTAT TAACC  30204
           |||||||||| |||||||| | |||||||||| |||||||||| | |||:|||| | |:|
122082   : CTTTACGTTC GACACCCATG TCGAGTAGAC CTCGTTCTTG TTAGAACTAT TCATC  122136
```

(III) Similarity : 93%
Length : 119bp
Repeat type : Direct-Antisense reverse repeat
Location : *rrn16*-**IGS**-*trnV-GAC* (from 107721 to 107603)
trnL-CAA-**IGS**-*ycf1* (from 121376 to 121493)

```
107721   : ATTTAGTAAA GAAAATGAAA GGATATTGAT AAACATCTTT CTTATAAGAA AGATATTATA
           ||||||||||  ||||||||| |||||||||| |||||||||| ||   : ||| |||||||:||
121376   : ATTTAGTAAA CAAAATGAAA GGATATTGAT AAACATCTTT CTATAG-GAA AGATATTGTA

           TAGATGATAG ATAATTCGTA AATCGACTTC GTCCACGAAG AAGGGAGCTA TAAGTAATA  107603
           |||||||||| |||||||||| |||||||||| |||||||||| |||||||||| ||||| | |
           TAGATGATAG ATAATTCGTA AATCGACTTC GTCCACGAAG AAGGGAGCTA TAAGTTAGA  121493
```

(IV) Similarity : 94%
Length : 108bp
Repeat type : Direct-Direct repeat
Location : *psbJ*-**IGS**-*clpP* (from 24231 to 24338)
ccsA-**IGS**-*petA* (from 131214 to 131321)

```
  24231 : TTAGGAAGGA AGAAGAGTCG GTATTATTGC CGGTAATAGA ATATTACTGG GGGAGAACCG
        : |||||||||| ||||||:||| |||||||||| |||||||||| |||||||||| |||||||:||
 131214 : TTAGGAAGGA AGAAGAATCG GTATTATTGC CGGTAATAGA ATATTACTGG GGGAGAATCG

          GAAACCCAGC AAAAAACAAA TCCAAATTTT TCGGAGACCT ATAGTTTC  24338
          |:|||:|||| |||||||||| |||:|||||| ||| ||| || | |||||:
          GGAACTCAGC AAAAAACAAA TCCGAATTTT TCGCAGAACT AAAGTTTT  131321
```
